# Design and Development of Micro-Power Generating Device for Biomedical Applications of Lab-on-a-Disc

**DOI:** 10.1371/journal.pone.0136519

**Published:** 2015-09-30

**Authors:** Karunan Joseph, Fatimah Ibrahim, Jongman Cho, Tzer Hwai Gilbert Thio, Wisam Al-Faqheri, Marc Madou

**Affiliations:** 1 Department of Biomedical Engineering, Faculty of Engineering, University of Malaya, Kuala Lumpur, Malaysia; 2 Centre for Innovations in Medical Engineering, Faculty of Engineering, University of Malaya, Kuala Lumpur, Malaysia; 3 Department of Biomedical Engineering, Inje University, Gimhae, South Korea; 4 Faculty of Science, Technology, Engineering and Mathematics, INTI International University, Persiaran Perdana BBN, Putra Nilai, Nilai, Negeri Sembilan, Malaysia; 5 Department of Biomedical Engineering, University of California Irvine, Irvine, California, United States of America; 6 Department of Mechanical and Aerospace Engineering, University of California Irvine, Irvine, California, United States of America; Università degli Studi del Salento, ITALY

## Abstract

The development of micro-power generators for centrifugal microfluidic discs enhances the platform as a green point-of-care diagnostic system and eliminates the need for attaching external peripherals to the disc. In this work, we present micro-power generators that harvest energy from the disc’s rotational movement to power biomedical applications on the disc. To implement these ideas, we developed two types of micro-power generators using piezoelectric films and an electromagnetic induction system. The piezoelectric-based generator takes advantage of the film’s vibration during the disc’s rotational motion, whereas the electromagnetic induction-based generator operates on the principle of current generation in stacks of coil exposed to varying magnetic flux. We have successfully demonstrated that at the spinning speed of 800 revolutions per minute (RPM) the piezoelectric film-based generator is able to produce up to 24 microwatts using 6 sets of films and the magnetic induction-based generator is capable of producing up to 125 milliwatts using 6 stacks of coil. As a proof of concept, a custom made localized heating system was constructed to test the capability of the magnetic induction-based generator. The heating system was able to achieve a temperature of 58.62°C at 2200 RPM. This development of lab-on-a-disc micro power generators preserves the portability standards and enhances the future biomedical applications of centrifugal microfluidic platforms.

## Introduction

In the past decade, various researchers have shown interest in developing biochemical- and biological/medical- based sensors in an effort to perform assay integration on the lab-on-a-disc platform, also known as the centrifugal microfluidic disc platform [1–3]. The lab-on-a-disc platform has potential to emerge as a miniaturized and automated portable diagnostic tool in point-of-care applications [4–7].

The increasing demand for biosensors and biomedical assay integration on the discs has led to the introduction of active modes, particularly pumping and valving, on centrifugal microfluidic discs [2, 8, 9]. An active mode is a condition whereby the centrifugal microfluidic disc is augmented with external effectors to enhance the ability of the disc to perform multistep fluidic sequencing in detection, analysis and biosensor integration. Examples of such enhancements are the delivery of thermal energy using thermoelectricity, halogen lamps or lasers to perform Polymerase-Chain-Reaction (PCR) amplification [1, 10, 11], wax-valves triggering [12–14], and fluidic transfer and pneumatic pumping [1, 2, 12, 15]. In certain applications, electrical interfaces are applied to the rotating discs. Examples include electrochemical-based glucose concentration measurements [16], sample enrichments using dielectrophoresis (DEP) [17] and electrochemical velocimetry biosensor integration to measure the fluid flow [18]. The above examples indirectly reflect the need for a power source on centrifugal microfluidic discs without negating the idea of portability and efficient energy/power management. The limitations of the enhancements made above are summarized in **Table 1**. Estimation of power consumption for each enhancement are also included in the table. From the Table 1, the most power consuming applications are the thermal-energy based applications. This is an important factor in developing the micro-power generators because it has the capability to replace the enhancement and perform similar function for the thermal energy applications. To prove this concept, we have introduce a novel method of localized heating technique on centrifugal microfluidic disc powered by the micro-power generators.

**Table 1:** Summary of energy-drawing applications on centrifugal microfluidic discs.

Energy Drawing Application on CD	Method	Estimated Power Consumption	Limitation
**Liquid Handling And Storage Using Waxes[14]**	Focused Infrared (IR) Radiation	75 Watt	Inefficient heating method
**Thermal-Pneumatic Liquid Pumping[12]**	Focused Infrared (IR) Radiation	75 Watt	Inefficient heating method
**Vacuum And Compression Valving Using Paraffin-Wax[13]**	Forced Convection Heat Transfer	2000 Watt	Convection heat transfer, wide area of heating
**Thermo-Pneumatic Push And Pull Liquid Pumping [15]**	Forced Convection Heat Transfer	2000 Watt	Convection heat transfer, wide area of heating
**Rapid Amplification PCR With Integrated Heating And Cooling[10]**	Thermoelectricity	1000 Watt	CD motion needs to be stopped to heat and freeze
**Integration Of Carbon-Electrode Dielectrophoresis [17]**	Dielectrophoresis	50 Watt	External connection using slip ring arrangement
**Whole Blood Analysis Utilizing Electrochemical [3]**	Electrochemical	50–200 Watt	Additional slip ring arrangement required
**Velocimetry In Monitoring Flow Real-Time [16]**	Electrochemical	50–200 Watt	Additional slip ring arrangement required

The micro-power generators are inspired by energy harvesting technology, which targets the energy derived from the rotational environment of a lab-on-disc system. **Table 2**summarizes the types of energy harvesting methods for power generation in rotational environments. Energy harvesting has been a focal point, especially in the field of wireless sensing and monitoring systems[19, 20]. Wireless systems have made it possible to place sensors in remote places or isolated systems, which eliminates issues related to cabling and makes a perfect fit for an area where continuous monitoring is crucial[19, 21–24]. Wireless sensor nodes are normally battery-powered, but battery replacement is not a favorable option for wireless network nodes [25, 26]. For that reason, the sensor nodes are designed to operate with an economical energy budget [27–29]. Current wireless sensor networks’ power requirements stand in the range of microwatts, which allows for the integration of alternative power sources for the sensors and eliminates the requirement for batteries to power up the sensors [30, 31].

**Table 2:** Summary of rotational energy harvesting methods and achievable power output.

Method	Piezoelectric	Thermoelectric	Photovoltaic	Electromagnet
**Wind Turbine Blades[22]**	*3*.*125μW*	*0*.*1 mW*	*235 mW*	*N/A*
**Rotating Propeller of a Ship[20]**	*N/A*	*N/A*	*N/A*	*80 mW*
**Rotational Generator on Spinning Machine[19, 23, 26]**	*N/A*	*N/A*	*N/A*	*10 mW*
**Energy Harvester on Rotating Wheel[35]**	*N/A*	*N/A*	*N/A*	*Few mW*
**Energy Harvester in Automobile Tires—Pulley System[36]**	*12 μW*	*N/A*	*N/A*	*N/A*
**Energy Harvester in Automobile Tires—Piezoelectric Patches[24]**	*70 μW*	*N/A*	*N/A*	*N/A*
**Piezoelectric Beams on Rotating Machinery[40]**	*7*.*7 mW*	*N/A*	*N/A*	*N/A*
**Piezoelectric Beams on Rotating Machinery—*limited self-tuning[41]***	*0*.*7 mW*	*N/A*	*N/A*	*N/A*

Micro fuel cells [32] and micro turbine generators [19, 33] are examples of alternatives that are based on chemical energy conversions [34]. Generating electrical energy from the surroundings of the sensor is an example of renewable energy harvesting[35, 36]. These alternative energy sources can be used directly or to supplement existing battery system to increase its longevity and the potential of the network [34, 37–39], depending on the application. This scenario is similar to the current progression in lab-on-a-disc research, wherein the integration of sensors in the disc requires cabling and enhancement. In the application of lab-on-a-disc, the abundance of rotational motion in the environment lends itself to the kinetic energy harvesting method. This paper highlights two methods to generate micro-power on a lab-on-a-disc and the detailed power output characteristics of both methods to provide a solution for interfacing power-driven applications directly on the microfluidic disc without any external fixation. As a proof of concept, a novel localized heating system was developed to demonstrate the effective application of power generated by the micro-power generators. This proof of concept also highlights the potential of the micro-power generators in solving one of the most power consuming application on centrifugal microfluidic discs (as detailed in Table 1).

### Energy Conversion Mechanism

Electrical power harvesting from the kinetic energy of rotational environments requires a transduction mechanism. Relative displacement or vibrations are converted to electrical energy by an electromagnetic, electrostatic or piezoelectric mechanism [30]. Electromagnetism results in current flow in a coil when there is relative motion between a coil and a magnet. Piezoelectric materials produce electrical potential when mechanical strain is applied to the material [30, 40, 41]. The relative displacement of two separated conductors by a dielectric (i.e., capacitor) causes a change in the energy stored in the capacitors. This is the electrostatic generation that converts mechanical motion to electrical energy. The disadvantage of an electrostatic convertor is that it needs another voltage source to start the conversion process. Because the use of an additional startup voltage source contradicts the fundamental goal of having a full self-reliant power generation system on the lab-on-a-disc system, only electromagnetic and piezoelectric conversion mechanism were examined on the lab-on-a-disc platform.

#### Piezoelectricity

A piezoelectric material becomes electrically polarized when subjected to mechanical strain. The polarization factor is directly proportional to the strain applied to the material [42, 43]. The type of available piezoelectric materials is numerous, however in this paper we will only focus on a specific polymeric material, Polyvinylidene Fluoride (PVDF). The piezoelectric response that generates electricity when pressure is applied is known as the direct piezoelectric effect, which can be modeled by the following linearized Eq (1) [43, 44]:

Direct piezoelectric effect:

$$D_{i}=e^{\sigma }{}_{ij}E_{j}+d^{d}{}_{im}\sigma_{m},$$ (1)

where vector ***D***_*i*_ is the dielectric displacement (N/mV), ***E***_*j*_ is the applied electric field vector (V/m), ***σ***_*m*_ is the stress vector (N/m^2^), ***d*** ^*d*^_*im*_ is the piezoelectric coefficients (m/V) and ***e*** ^*σ*^_*ij*_ is the dielectric permittivity (N/V^2^). Superscript ***d*** indicates that the term is for the **direct effect**.

**Fig 1:**
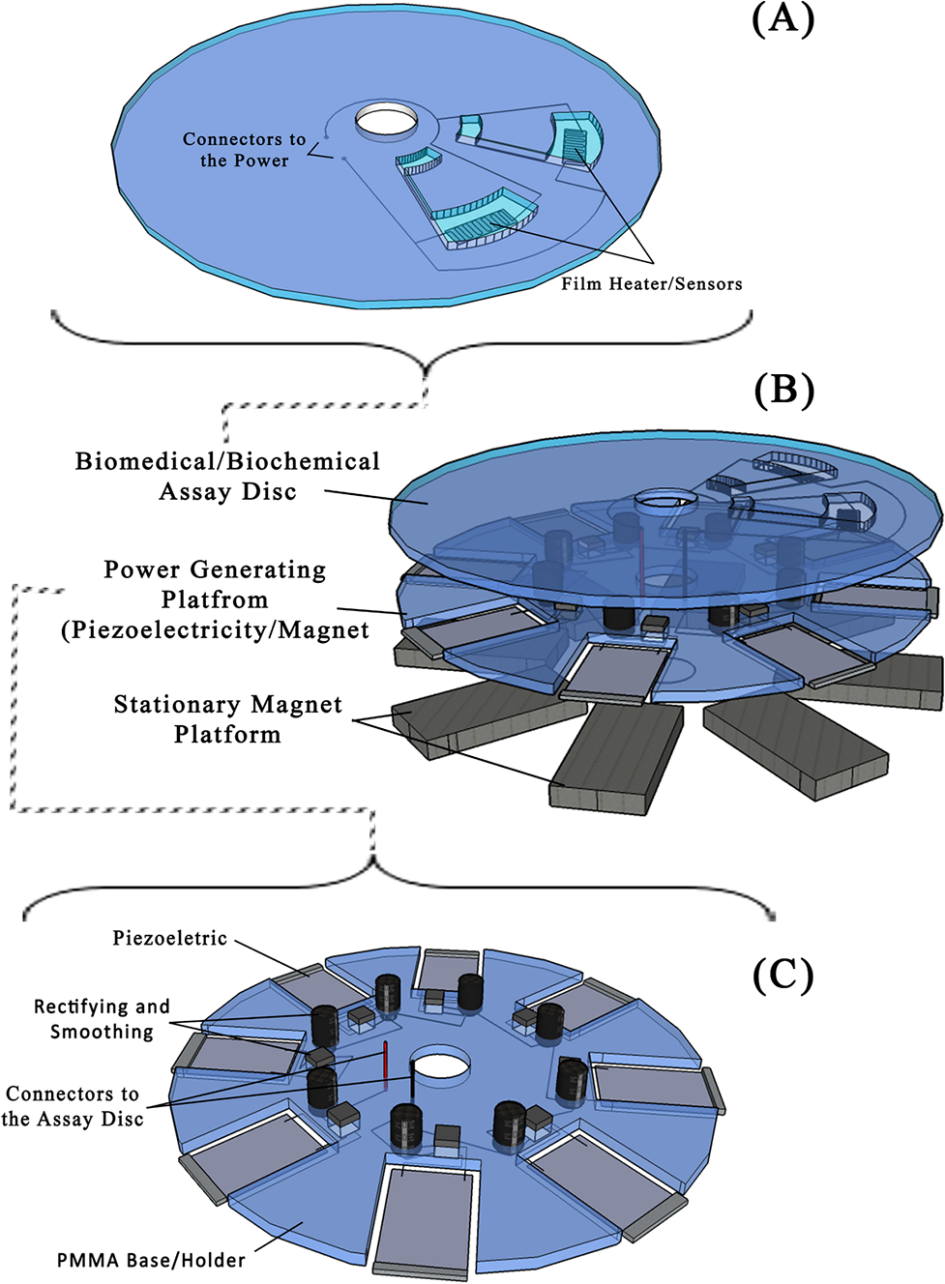
(A) The experimental arrangement of the piezoelectric film-based power generation system. (B) An example of a thermal energy-drawing disc that will be placed on the top of the power generating disc. The power from the power generation disc will be delivered through the power connectors and the heater pad will convert the electrical energy to thermal energy for a possible biomedical assay to take place. (C) The piezoelectric film-based power generation disc illustration. The outputs from the piezoelectric film are coupled to a rectifier and a capacitor to rectify and smooth the output power.

Goldfard *et al*. concluded that piezoelectric materials achieve higher efficiency at frequencies within the resonant frequency of the material [42, 43, 45, 46]. The concept of piezoelectric film power generation system on the lab-on-a-disc platform is illustrated in Fig 1.

#### Electromagnetic induction

**Fig 2:**
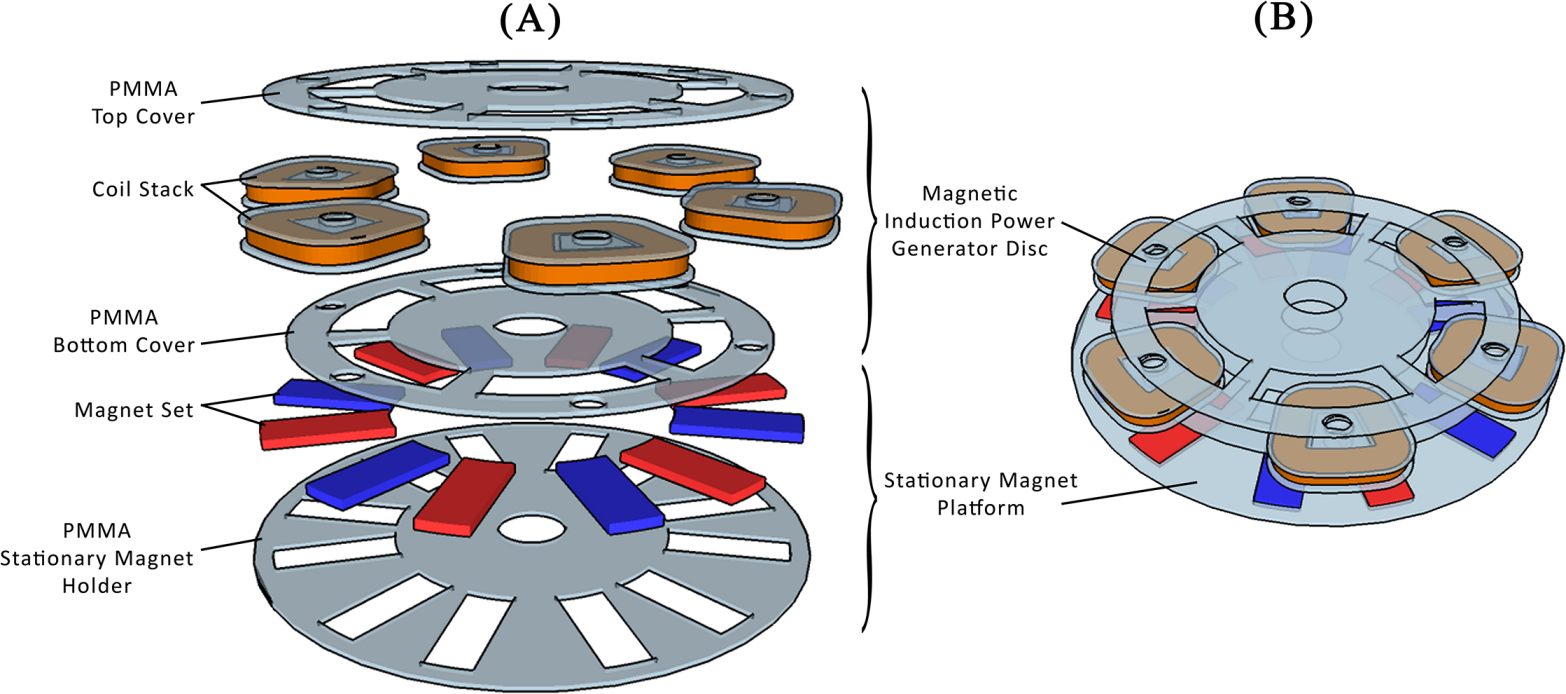
(A) The expanded view of the magnetic induction-based power generation disc. (B) The experimental arrangement of the magnetic induction based power generation system with the stationary magnet platform on the bottom, the power generation disc on the rotating platform along with the energy-drawing disc.

In our application, we reproduce the effect of a fluctuating magnetic field to generate current in the coils placed on the power-generating discs. The power-generating disc is sandwiched between the centrifugal microfluidic disc (where the biosensors are placed) and the stationary magnet platform (embedded on the spinning platform). When the disc rotates, there is a fluctuating magnetic flux passing through the coils. The power-generating disc and centrifugal microfluidic disc with biosensors will have an electrical connection and spin at the same time. The conceptual figure of the system is illustrated in Fig 2 and the voltage output of the coil can be generalized with the following equation:

$$V\;=NA\;\frac{dB}{dt}\;\text{sin}\;\alpha ,$$ (2)

where ***V*** is the induced voltage, ***N*** is number of turns of the coil, ***B*** is the magnetic field provided by the stationary magnets, ***A*** is the area of the coil, and $\frac{\mathrm{dB}}{\mathrm{dt}}$ denotes the rate of magnetic field change. The angle of the magnetic field direction to the coil plane is denoted by ***α*** [46, 47]. In our application, the rate of magnetic flux change is related to the rotational rate applied to the power generation disc, and ***α*** is 90°. The correlation of the applied spinning speed and the frequency of voltage generated from the coil are detailed in the **Results and Discussion** section.

In this paper we introduce two systems of independent power generation for electrically dependent biosensors on centrifugal microfluidic discs. This paper details the prototype model of the power generating discs, the fabrication and the power output analysis of the system. A novel localized heating system, powered by the micro-power generators was developed as a proof of concept to the independent power generation system.

## Power Generator Disc Design and Fabrication

### Piezoelectric Film Disc

**Fig 3:**
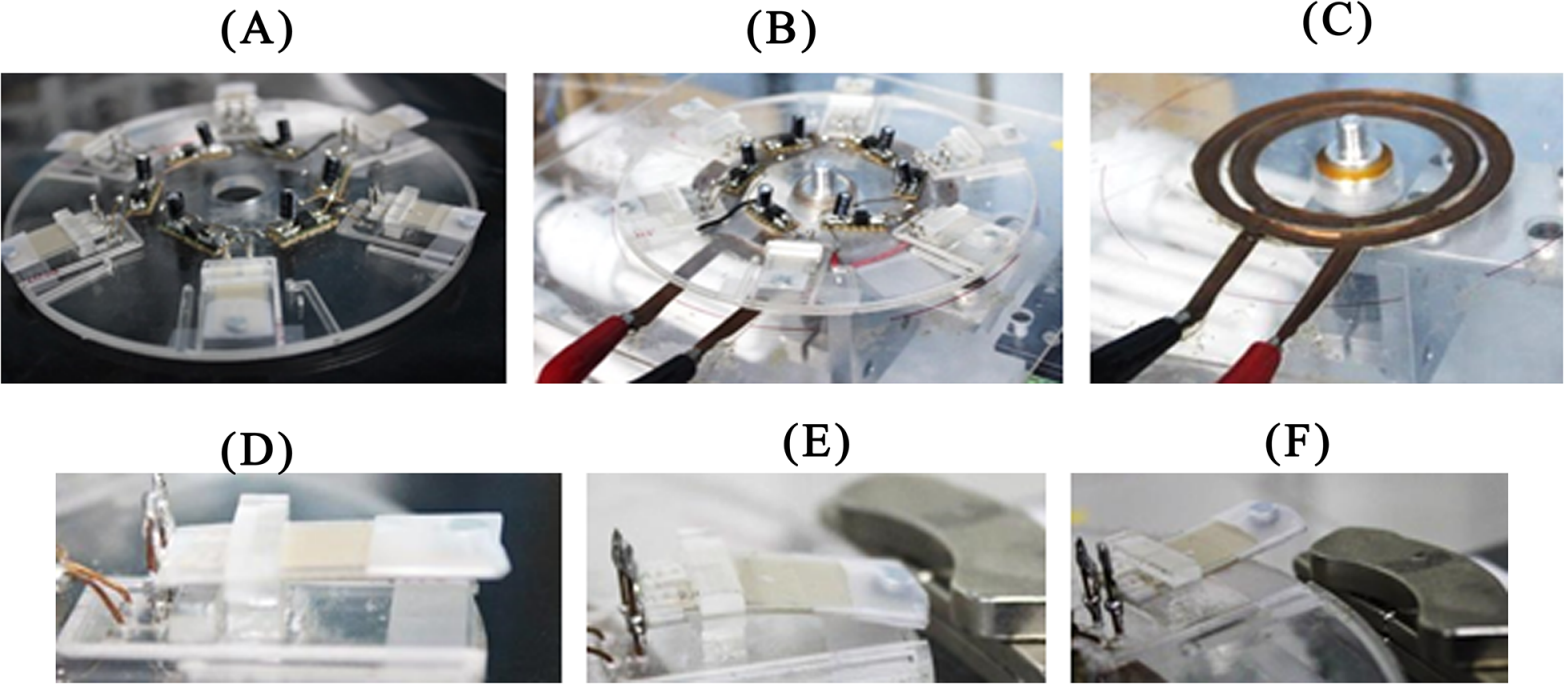
(A) The fabricated piezoelectric film-based power generation disc. (B) The experimental setup of the piezoelectric-based power generation system. (C) The stationary copper plate on bottom of the power generator disc, which was used to read the output from the power generation disc. (D–F) The deformation of the piezoelectric film when introduced to a stationary magnet. This is the introduced vibration to enhance the deformation. The rate of the deformation is manipulated by varying the speed of rotation of the power generating CD.

Piezoelectric film-based power generating discs are made of 3 layers: 2 layers of 2 mm Polymethyl Methacrylate (PMMA) and one layer of Pressure Sensitive Adhesive (PSA) material (FLEXcon, USA). These are used to construct the platform for the piezoelectric film (FS-2513P - PROWAVE) slots. Each rectifying circuit was implemented with a Surface Mount Device (SMD)-type bridge rectifier (HD01-T by DIODES INC.) with a 22 μF capacitor (by RUBYCON) to rectify and smooth the output voltage from each piezoelectric film. The rectifying circuits were placed on the disc (see Figs 1C and 3A), and the piezoelectric film’s electrodes were connected to the bridge rectifier (Fig 3).

### Electromagnetic Induction Disc

**Fig 4:**
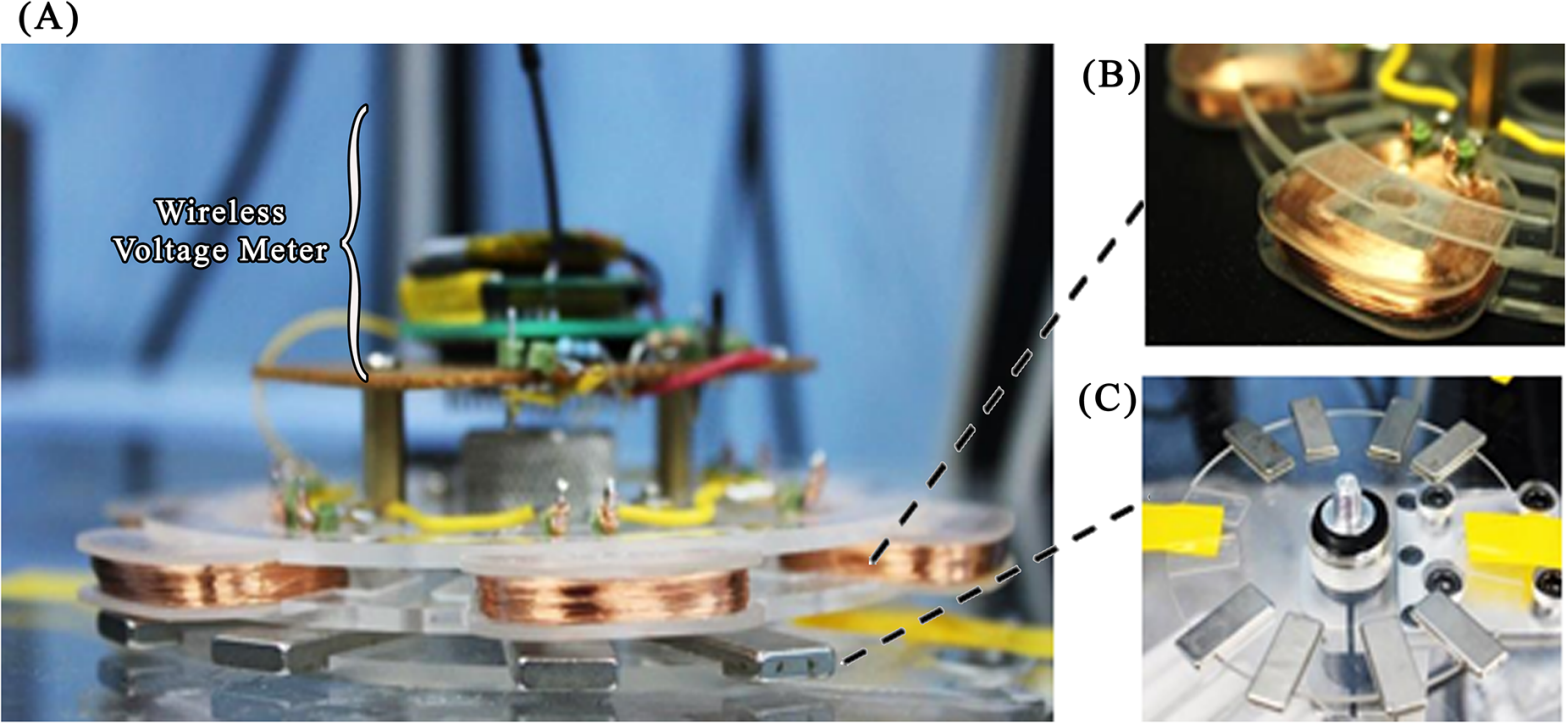
(A) The experimental arrangement of the magnetic induction-based power generation system. The stationary magnet platform on the bottom and the wireless voltage meter on the top transmitted the output voltage generated from the power generation disc. (B) The coils used in the power generation disc. (C) The stationary magnet platform placed on the bottom of the power generator disc.

An electromagnetic induction-based self-generating power disc was made of 6 stacks of coils with two layers of PMMA to clamp the coils. Geometrically, adjacent coils were placed at an interval of 60° with respect to the center of the disc. The stationary magnets placed on the spinning platform were arranged following the geometry of the coils. Each coil stack was made of 1100 turns of enamel-insulated copper wire AWG-38 (Fig 4B). Each coil stack is paired with two stationary magnets with opposite polarity as shown in Figs 2 and 4.

### Power Generation Protocol

A centrifugal microfluidic disc spin test platform consists of a motorized module attached to a solid platform and controlled by computer software [13, 15, 48]. For the piezoelectric film power generator disc experiment, a layer of 2 mm thick PMMA was attached with two rails of copper sheets (Fig 3C) placed on the bottom of the disc to read the voltage output from the power generator disc. For the electromagnetic induction disc experiment, a platform with a stationary magnet (Fig 4C) and a wireless voltage meter were used.

#### Piezoelectric film disc

A preliminary experiment was devised to investigate the effect of the resonant frequency of the piezoelectric film to generate maximum electrical power output. In order to perform this experiment, a vibration system was designed. The system consists of soft iron poles that are magnetically induced to switch polarity following the frequency set by a function generator (GWINSTEK-GFG-8255A). A piece of ferromagnetic material was attached to the end of the piezoelectric film and placed between the two soft iron poles. The peak-to-peak voltage (Vpp) generated at each 5 Hz step from 0–120 Hz was recorded. The same step was conducted with the resistive load connected across the output terminals of the piezoelectric film. The load resistors used were 10 kΩ, 100 kΩ, 1 MΩ, and 10 MΩ.

For testing the power generation capability of the film, the centrifugal microfluidic-like disc was fitted with six piezoelectric films. The centrifugal microfluidic-like disc was then placed on the motorized disc spin test platform to mimic the real spinning condition. The output terminal of each film was connected to the rectifying and smoothing circuit. In order to synchronize power generation with the spinning speed, the film’s fluctuation was induced by a tiny magnet on the edge of the film and a bar magnet on the stationary platform (Fig 3D–3F). The tiny magnet was placed at the edge of the film to increase the deformation of the film. The stationary platform has rails made of copper that provide an electrical connection between the piezoelectric film disc and the oscilloscope for monitoring the output voltage when the disc is spinning (see Fig 3C). The disc was spun from 0 to 800 RPM at increments of 100 RPM every 300 seconds.

#### Electromagnetic induction disc

For this experiment, a wireless voltage meter is used to measure the output voltage from each coil. The reading is transmitted to a computer in real time. At first, a pair of magnet bars was placed on the stationary platform in opposite polarity position facing towards the coil at 90° to the coil’s plane. The frequency of voltage generated from the coil was monitored to be proportional to the spinning rate of the power generator disc. The phase angle difference was also monitored by capturing waveforms of the voltage outputs from the first and second coils simultaneously and then with the first and third, and with the first and fourth.

#### Localized heating technique with wireless temperature monitoring

**Fig 5:**
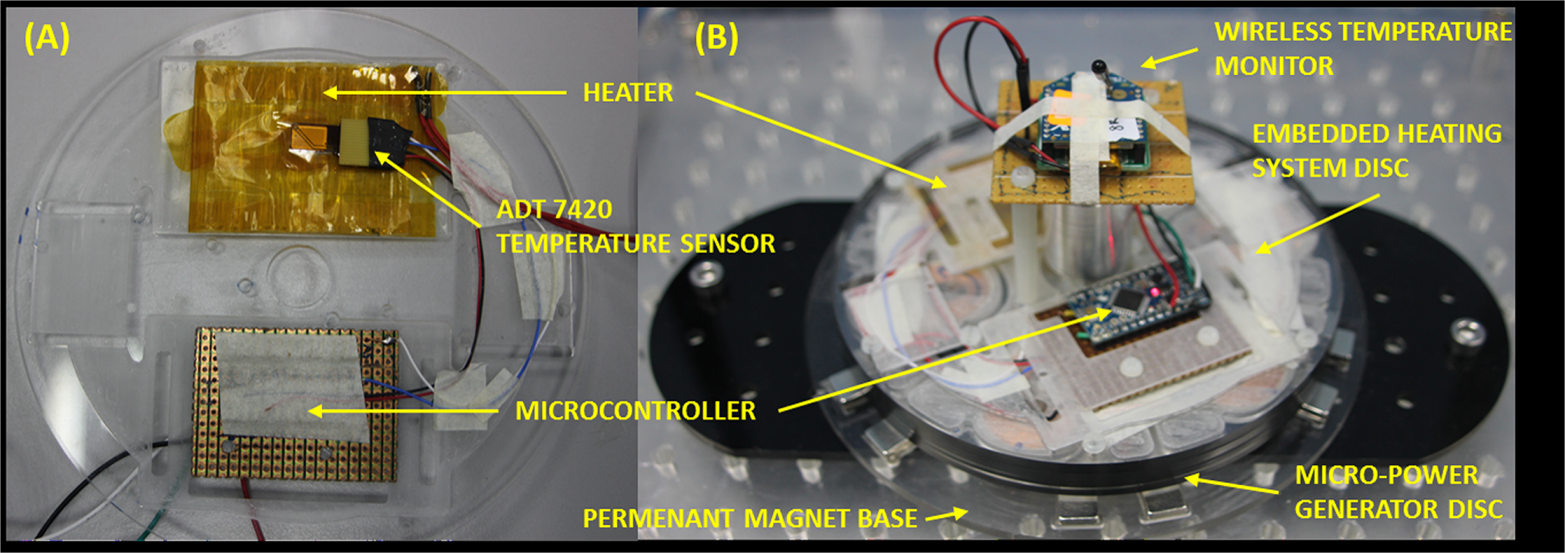
The modular heating disc configuration and the integrated wireless temperature module.

To demonstrate the capability of the proposed micro-power generator, localized heating on the microfluidic CD was implemented in this work. A heating element was implemented to heat specific areas of the platform. In this experiment, a modular heating disc consisting of a heating element and wireless temperature monitor was stacked on top of the micro-power generator disc (Fig 5). The circuits on the two discs were connected using insulated 18 American Wire Gauge (AWG) copper wires (Fig 5B). The heating element was made from 40 AWG Nickel Chromium wire with resistance of 555 Ω, woven on a Pressure Sensitive Adhesive (PSA) film (Fig 5A). The heating element was electrically insulated with Kapton film which is able to withstand high temperature. To monitor the temperature change caused by the heating element, a digital temperature monitoring system was embedded onto the platform. The temperature monitoring system consists of an ADT7420 digital temperature sensor connected to an ATMEGA328PU microcontroller. Temperature readings were sampled every 240 milliseconds by the ADT7420 and sent to the microcontroller using Two-Wire-Interface (TWI) protocol. The microcontroller the processes the readings and wirelessly-transmits the temperature data to a remote temperature monitoring computer using a pair of ZigBee modules (see Fig 5B). A lithium polymer battery was used to power the microcontroller and the ZigBee module to ensure continuous data transmission. Temperature data received by the remote computer were monitored and recorded using RealTerm terminal program.

## Results and Discussion

### Piezoelectric Film Disc

**Fig 6:**
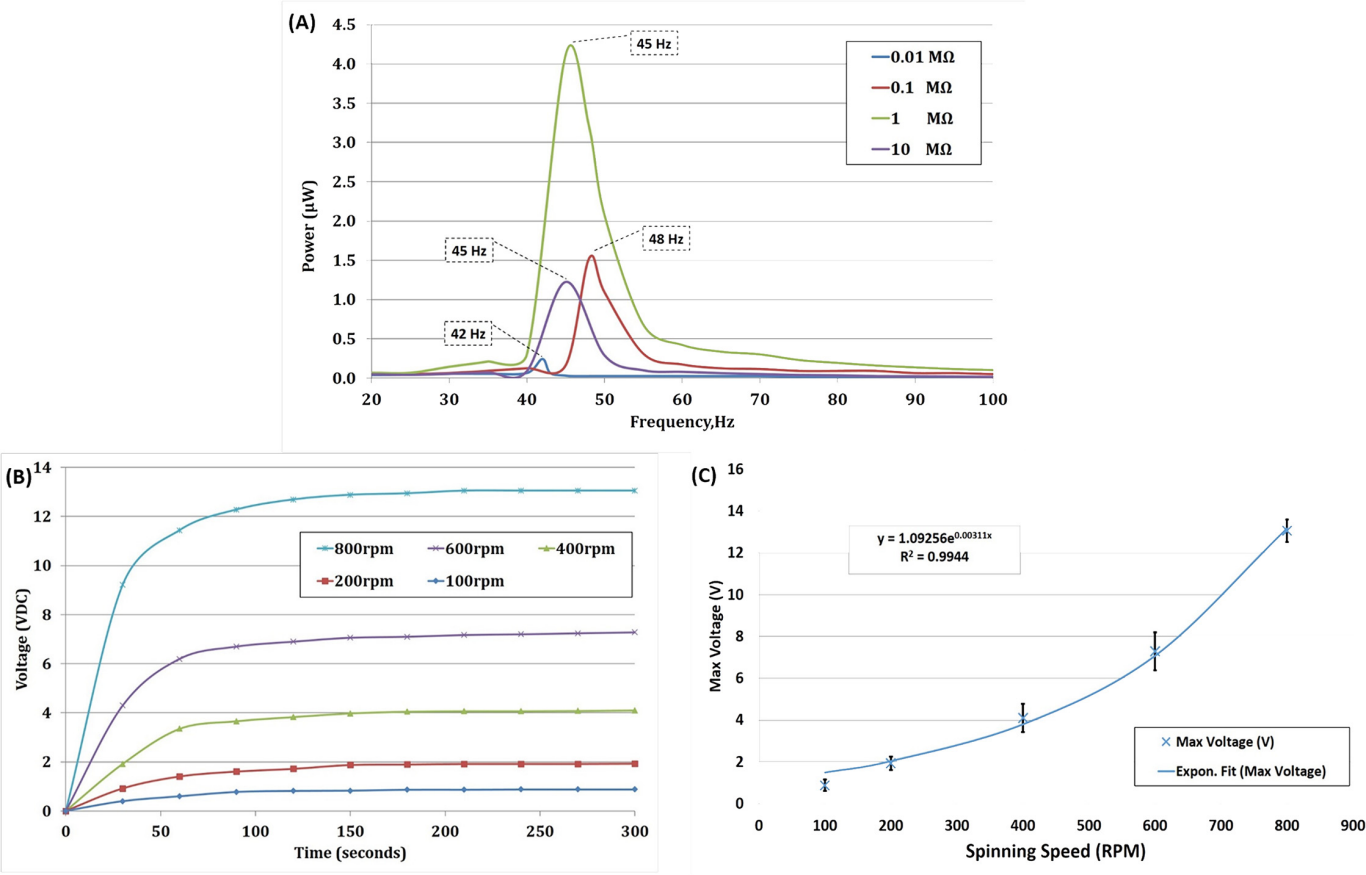
(A) The output power at different frequencies of vibration and resistive load. The frequency when the maximum power measured is highlighted in the dotted box. (B) On the CD test: Variation of voltage output with spinning speed (RPM) for 300 seconds. No load applied to the system (C) On the CD test: Maximum voltage generated at each particular spinning rate (RPM). The solid line represents exponential fit to the experimental data with goodness of fit up to 0.9944 (n = 5).

The piezoelectric film generates AC voltage as a result of the periodic vibration (which is directly proportional to the spinning speed). From Fig 6A, we can clearly conclude that the average maximum power (in μW) of all four piezoelectric films tested occur during vibration frequencies between 42–48 Hz. This correlates with the hypothesis of Goldfarb *et al*. [45], which concluded that the maximum voltages are observed within the range of the film’s resonant frequency. It can also be deduced from the result shown that the resonant frequency of the piezoelectric films used in the experiment were in the range of 42–48 Hz. The effect of the resistive load on power generation is also shown in Fig 6A. A resistive load of 1 MΩ provides optimum power generation compared to other loads. This is consistent with the maximum power transfer theorem as it is closest to the piezoelectric film’s impedance at resonance [49, 50]. The variation observed suggests that the development of a modular piezoelectric power generation system depends on two factors: (i) the load applied to the system needs to be matched with the internal impedance of the system, and (ii) the vibration frequency range applied to the system need to be between 42 to 48Hz.

Knowing the resonant frequency and internal impedance of the piezoelectric film helps in determining the spinning speed needed to generate maximum power output. A piezoelectric film based micro power generator was devised to be integrated with the real centrifugal microfluidic setup as shown in Fig 3. Due to the limitation of the spinning system, the maximum spinning speed for the piezoelectric film based power generator was 800 RPM (13.33 Hz). Since the maximum spinning speed is lower than the resonant frequency range of the piezoelectric film, the power generation was insignificant (refer Fig 6A). Therefore, the voltage generation was observed in this experiment. Voltage output for each spinning speed was observed and recorded for 300 seconds as shown in Fig 6B. An increase in spinning speed results in an increase in the voltage generated by the piezoelectric film. There also exist a transient settling time for the film to reach a stable output voltage which is caused by the capacitance of the piezoelectric film. In general, PVDF has a high dielectric constant compared to other polymers. In addition, the capacitance increases when the thickness of the film decreases and surface area of the film increases.

Fig 6C illustrates the generated voltage for five spinning speed points. An exponential fitted curve using least square method is also shown in the figure. It can be seen that the generated voltage is exponentially related to the rotational speed of the platform. In our experimental system, vibration frequency corresponds to the spinning speed of the centrifugal microfluidic platform. Increments in the spinning speed increases the voltage generated as it approaches the film’s resonant frequency. The output voltage (in DC) obtained from six rectifiers that were connected in series results in 13.4 V generated at a spinning speed of 800 RPM (13.33 Hz). In order to increase the vibration frequency while maintaining the spinning speed at 800 RPM, another stationary magnet was added exactly at the opposite position of the previous stationary magnet. This additional magnet was intended to introduce an additional deflection of the film per revolution. Theoretically these will double the vibration frequency factor. However, the experiment was not successful due to the spinning system’s limitations. Attraction force developed between the stationary magnet and the on-films magnets results in a vertical force on the disc’s edge whenever the magnets come into contact. This develops an extra mechanical load to the spinning system, which eventually stops the spinning due to the low torque capacity of the spinning system motor.

### Electromagnetic Induction Disc

An electromagnetic induction system provides a number of possible manipulations to enhance the output. Example of possible variations includes the number of turns, the thickness of the coil, the area exposed to the magnetic field and the magnetic field density provided by the stationary magnets. An experiment was performed to observe the potential output power generation with two coils of different thickness and number of turns. The magnetic flux rate (0.47 mWb/s) was made to be constant for both coils. The results are shown table in **Table 3**.

**Table 3:** Power generated with different number of turns that is contributed by the diameter of the coil.

Coil Diameter (mm)	No of Turns*	Resistive Load (kΩ)	Voltage (Vrms)	Current (mAmp)	Power (nW)
**0.2**	300	1	0.144	0.144	20.736
**0.5**	80	1	0.056	0.056	3.136

*Note: Influenced by the diameter of copper coil used because the total space for coil on the disc is fixed.

**Fig 7:**
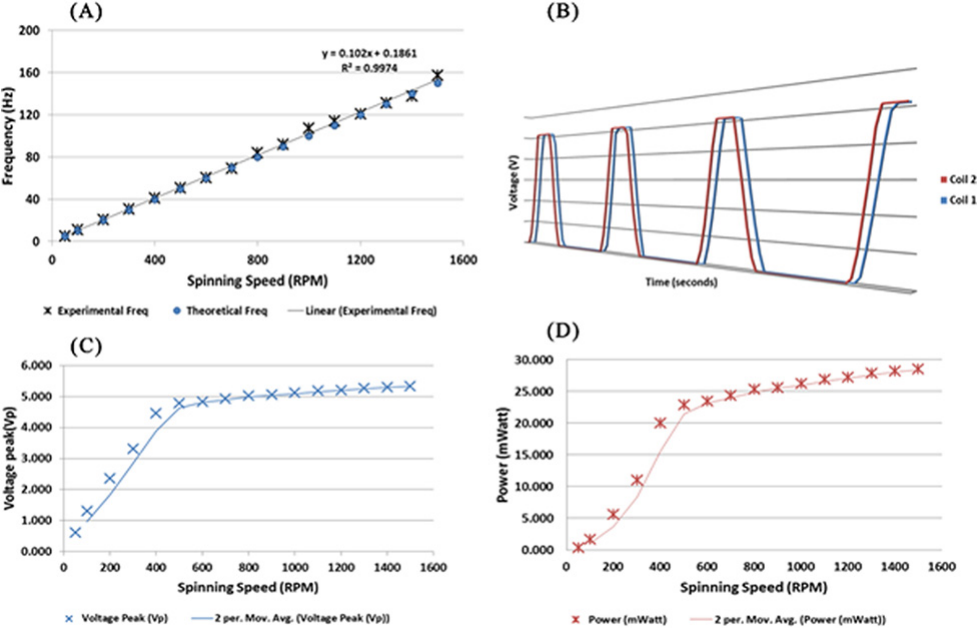
(A) Frequency of the voltage generated in comparison with the calculated frequency from the spinning speed of the disc. The black line represents the linear fit of theoretical and experimental data with a linear regression value of R^2^ = 0.9974 (n = 16). (B) Generated voltage from two coils adjacent to each other with a 60° angle in between. 3D perspective chosen to represent the graph is to show the very minute phase difference between both coils’ outputs, approximately 1.016 ms. (C) The peak voltage generated from a single coil relative to the spinning speed (RPM). Output peak voltage illustrated by the moving average trend line of 2 periods. (D) Power generated relative to the spinning speed and the trendline illustrated with the moving average of 2 periods.

The operational principle for this electromagnetic induction system is to control the power output by varying the spinning speed of the disc. In the experiment, the spinning speed contributes to the magnetic field change $\left(\frac{\mathrm{dB}}{\mathrm{dt}}\right)$ over time. The spinning speed contributes to the rate (ω) of the coil exposed to the “magnetic field”. The correlation of the spinning speed and the output voltage frequency are shown in Fig 7A. The generated output frequency using the six pairs of magnets results in six times the applied spinning frequency.

The coils on the power generator disc had 60° between one another from the disc’s center. The magnets were arranged in pairs of north-pole and south-pole following the arrangement of the coils on disc (Figs 2 and 4). Fig 7B illustrates the waveform generated from 2 adjacent coils. The two coils were placed adjacent to each other (60° in between) and the output voltage from each coil was measured simultaneously. Similar outputs were observed even for coils with 120° and 180° in between. There was a phase difference of 1.016 ms between the two measuring channels caused by the Analog to Digital Conversion (ADC) channel switching frequency of the wireless voltage meter.

The power generation experiment was conducted to evaluate the power a single coil could generate at 1500 RPM. A voltage divider was used to measure voltage above the reference voltage of the voltage meter and also works as a resistive load to the system. The power generated with a load of 1000 ohm is shown in the Fig 7C and 7D. The graph shows a higher uptrend gradient until 500 RPM and the slope’s gradient decreases. The higher gradient portion agrees with **Eq 2**as the rate of magnetic flux change increases. The decrease of the voltage and power generation gradient from 600 RPM to 1500 RPM is caused by the current generated in the coil exceeding the capacity of the enamel wire. This can be justified as heat was observed from the coils during the experiment.

### Piezoelectric Film Disc *vs*. Electromagnetic Induction Disc Power Generation Characteristic

Both methods introduced have different ranges of power generating capabilities and a certain number of adjustable parameters to suit the biosensor characteristics and applications dynamics. Thermal energy-drawing applications would require a higher range of power capacity; therefore, an electromagnetic induction system would be more suitable. Electrochemical detection systems would require a high voltage and very small current system in order to maintain the electrodes potential. This can be addressed by both piezoelectric film and electromagnetic induction systems with power/charge management instrumentation applied. A piezoelectric film-based system needs a high spinning speed (2400 rpm) to reach its theoretical resonant frequency range to achieve higher voltage generation. Fluctuations of the generated voltage of the piezoelectric film caused by irregular deformation are crucial. Thus, rectification of the output from the system is needed to provide a stable DC voltage source. Meanwhile, for an electromagnetic system, the AC voltage generation is stable and also possible to be rectified for DC voltage based biosensors and application. In terms of adjustable parameters, a piezoelectric film-based power generation system lacks the advantage of configurability because the parameters are tightly correlated to the piezoelectric film’s own characteristics. The vibration achieved by increasing the spinning rate (rpm) or the number of deflection points discussed in above. In contrast, the electromagnetic induction system has better configurability advantages. The desired voltage or power could be generated by adding or removing coils or the number of turns in each coil. In addition, the strength of the magnetic flux density can also be varied by adjusting the distance between the power generating platform and the stationary magnet platform (Fig 4). In order for the spinning rate not to conflict with the microfluidic sequences on the centrifugal microfluidic disc, the power generation needs to be activated at a low spinning rate. In that context, the electromagnetic induction-based power generation is a better option for biosensor applications on the disc.

### Application of Micro-Power Generator Disc for Localized Heating Using Embedded Heating System on CD

Many potential applications can be implemented using the power generated by the proposed micro-power generators. In this section, localized heating that is useful in many mechanical and biomedical applications such as liquid pumping and PCR is demonstrated with the micro-power generators. As a reference for this demonstration, a summary of existing applications that require heating (require energy) is presented in Table 4.

**Table 4:** Temperature requirements of current thermal energy-drawing applications on the CD.

Thermal Energy Drawing Applications	Temperature	
	Minimum	Maximum
**Liquid Handling And Storage Using Waxes[14]**	51.6°C	62.7°C
**Thermal-Pneumatic Liquid Pumping[12]**	25°C	57°C
**Vacuum And Compression Valving Using Paraffin-Wax[13]**	26°C	57.2°C
**Thermo-Pneumatic Push And Pull Liquid Pumping[15]**	27°C	50°C
**Rapid Amplification PCR With Integrated Heating And Cooling[10]**	60°C	95°C

**Fig 8:**
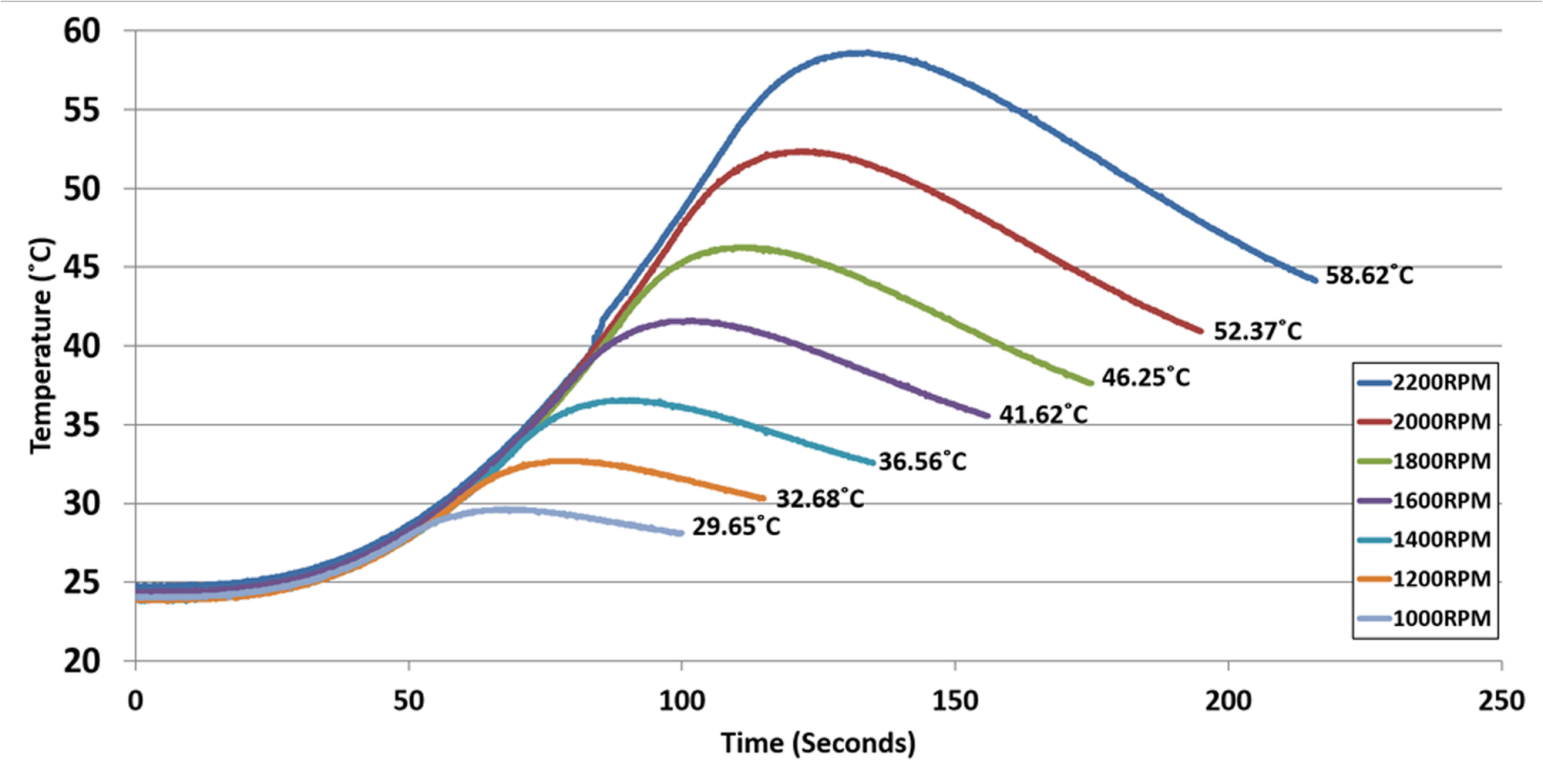
Shows the heating gradient based on the maximum spinning speed. The maximum temperature for each graph is denoted on the graph. The experiment was done in a closed chamber fixed on top of the spin test system. The micro-power generator used in this experiment is made of 6 coil stacks. Each coil stack was made of 1100 turns of enamel-insulated copper wire AWG-38.

To aid in future implementation of the power generator for heating applications, a study on the achievable temperature at various spinning speeds is performed. A power and a heating discs were stacked together (see Fig 5B) and were mounted on the spin test system. The discs were spun at increasing spinning speeds (at increments of 100 RPM per 5 seconds) till maximum speeds ranging between 1000 and to 2200 RPM. Once the discs reach the intended spinning speed, the speed is then gradually reduced to 0 RPM (at decrements of 100 RPM per 5 seconds). Prior to the start of each heating cycle, the discs were left to cool down to the ambient temperature of 25°C. Fig 8 shows the achievable heating element temperature in the disc at different spinning speeds. Based on the results, the embedded heating system is able to heat up to a temperature of 58.62°C in 130 seconds at a maximum spin speed of 2200 RPM. This temperature meets the requirement of wax valving and pneumatic pumping applications on the CD (see Table 4), but at a much shorter heating time. Note that as the disc speed is reduced, the power supplied to the heating discs is reduced, resulting in less heat generated by the heating disc. Concurrently, as the disc is continuously air cooled at room temperature when spun, at higher temperatures the temperature drops more significantly (after reaching the maximum spin speed) due to the bigger variance between the disc temperature and the ambient temperature [15]. From Fig 8, it is also observed that the achievable temperature increases with higher spinning speeds. This shows that the system can produce heat at a high range of temperatures to suit the various applications on the CD. However, in order to perform a more controlled and automated application, the heating and also temperature monitoring systems require further improvements. This includes a power management module, a temperature controller, and full-duplex communications to enable wireless control and monitoring of the system (the current system is an only half-duplex, and only allows temperature monitoring).

## Conclusion

We have introduced two methods of micro-power generators for biosensors integration on-disc and energy drawing applications with two different ranges of power generation capability. Current disc-based applications that require electrical energy still rely on the conventional method of transferring the electrical energy through an electrical interface on the spin rotor chunk connected to the discs [16, 17]. This idea conflicts with the initial conception of centrifugal microfluidic technology to be independent from external peripherals or power connections, to be fully portable and to consume a low amount of energy [4]. In order to meet the demand of complex biomedical point-of-care diagnostic systems, a micro-power generating disc application is introduced. This micro-power generator disc has been realized in prototype mode and holds numerous possibilities for advancing biosensor applications and centrifugal microfluidic technology. The findings indicate that the piezoelectric film-based power generation system managed to produce up to 24 μWatt at 800 RPM. The limitation of this method is the range of power it can possibly generate. The electromagnetic induction-based power generation system is able to generate up to 25 milliwatts for each coil at 800 RPM. The micro-power generator will be used as a portable power source to be coupled with the normal microfluidic disc, which needs power to run biomedical assays. The localized heating system, powered by the micro-power generators illustrates the capability of the system to perform heating on the centrifugal microfluidic disc. The proof of concept tackles the most power consuming application on CD and potential to be the solution for all other power-dependent application on CD. The system differs from the method of normal battery insertion because it promises a wide range of power outputs and is able to generate programmable outputs if coupled with a microcontroller. For example, to perform dielectrophoresis on disc, a function generator can be integrated with the micro-power generators to provide a programmable frequency output, depending on the application. Another application of the micro-power generator would be chamber-specific heating using film heaters directly embedded in the microfluidic disc.

The availability of an independent power source will motivate researchers to integrate more biosensors on centrifugal microfluidic discs. This will lead to a whole new point-of-care diagnostic system with higher throughput, based on the biosensor’s characteristics. A diagnostic system with biosensors multiplexed fluidic sequencing and wireless data transmission to laptops is possible with the introduction of this power generator on disc. In addition to biosensor integration, the micro-power generators can also work as an independent power source for any active mode applications on the centrifugal microfluidic platform. For example, the power generator disc introduces the possibility of a conduction-type heat transfer mechanism, which is better in terms of energy efficiency than the currently popular radiation and convection type heat transfer mechanism in the centrifugal microfluidics field[12, 14, 15]. The generated energy can be controlled by using the means of the spinning rate, which increases or decreases the frequency of vibration (piezoelectric film) or rate of magnetic flux exposure (electromagnetic induction). This will help the user to manipulate or control the desired power of the system. A complete independent power source system with a variety of power options is one of our main research objectives upon which we are currently focusing. In conclusion, the introduction of micro-power generators on lab-on-a-disc will lead to new avenues for biosensors and centrifugal microfluidic discs in biomedical applications.
